# Molecular MRI of myocardial peroxidase activity in ischemic injury reveals a chemical milieu incompatible with stem cell survival

**DOI:** 10.1186/1532-429X-18-S1-O16

**Published:** 2016-01-27

**Authors:** Howard H Chen, Y Iris Chen, Christian T Farrar, Eric M Gale, Peter Caravan, Ronglih Liao, John W Chen, David E Sosnovik

**Affiliations:** 1 grid.32224.350000000403869924Martinos Center for Biomedical Imaging, Department of Radiology, Massachusetts General Hospital, Harvard Medical School, Boston, MA USA; 2 grid.32224.350000000403869924Cardiovascular Research Center, Cardiology Division, Massachusetts General Hospital, Harvard Medical School, Boston, MA USA; 3 grid.62560.370000000403788294Cardiac Muscle Research Laboratory, Divisions of Cardiology and Genetics, Brigham and Woman's Hospital, Harvard Medical School, Boston, MA USA; 4 grid.32224.350000000403869924Center for Systems Biology, Massachusetts General Hospital, Harvard Medical School, Boston, MA USA

## Background

The delivery of stem cells to the myocardium after ischemic injury has the potential to prevent left ventricular remodeling and heart failure. Ischemic injury, however, elicits a robust inflammatory response and the release of cytotoxic substances from infiltrating leucocytes. We aimed here to use a myeloperoxidase activatable gadolium chelate (MPO-Gd) [1], to quantify peroxidase activity in the myocardium *in vivo* after ischemic injury. We further aimed to determine, using bioluminescence imaging, whether the detected levels of MPO were compatible with cell survival.

## Methods

T1 mapping was performed in mice (n = 5) at 9.4T 24 hours after occlusion of the left coronary artery. The mice were injected IV with 0.2 mmol/kg of MPO-Gd and imaged for 2 hours. An ECG gated Look-Locker sequence was used with the following parameters: FOV 2.5 cm, matrix 160 × 160, slice 1 mm, flip angle 20 degrees, TR 3s, TE 1.5s, 4 averages, TI increment = RR interval. T1 maps (Matlab) were converted to relaxation rate (R1) maps and the relaxivity (r1) of MPO-Gd at 9.4T was used to convert these into Gd-MPO concentration maps. MPO activity maps were subsequently derived based on the kinetic properties of the enzyme and probe. Bioluminescence imaging (BLI) of luciferase-expressing cardiac side population progenitor cells was performed with the cells exposed to the range of MPO concentrations detected *in vivo.* Serial BLI of mice injected with cells on day 0 (n = 7) or day 14 (n = 7) after ischemic injury was performed.

## Results

**Figure 1:**
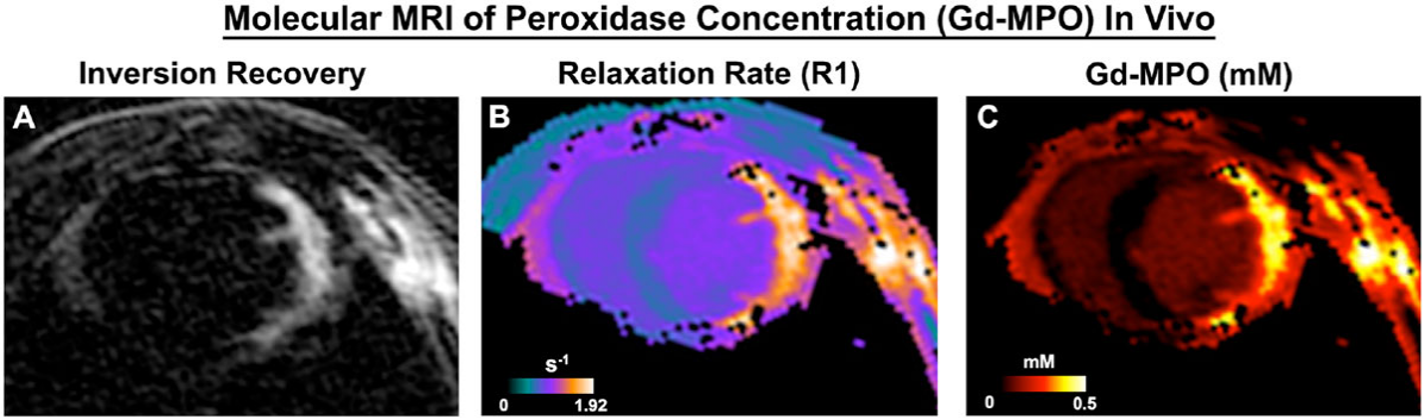
**Molecular MRI of Gd-MPO activation in vivo**. **A.** Inversion recovery image 2 hours after probe injection. Unbound Gd has been washed out but activated probe is retained in the ischemic myocardium. **B.** R1 map of the same slice. **C.** Quantitative map of Gd-MPO concentration and activity in vivo.

**Figure 2:**
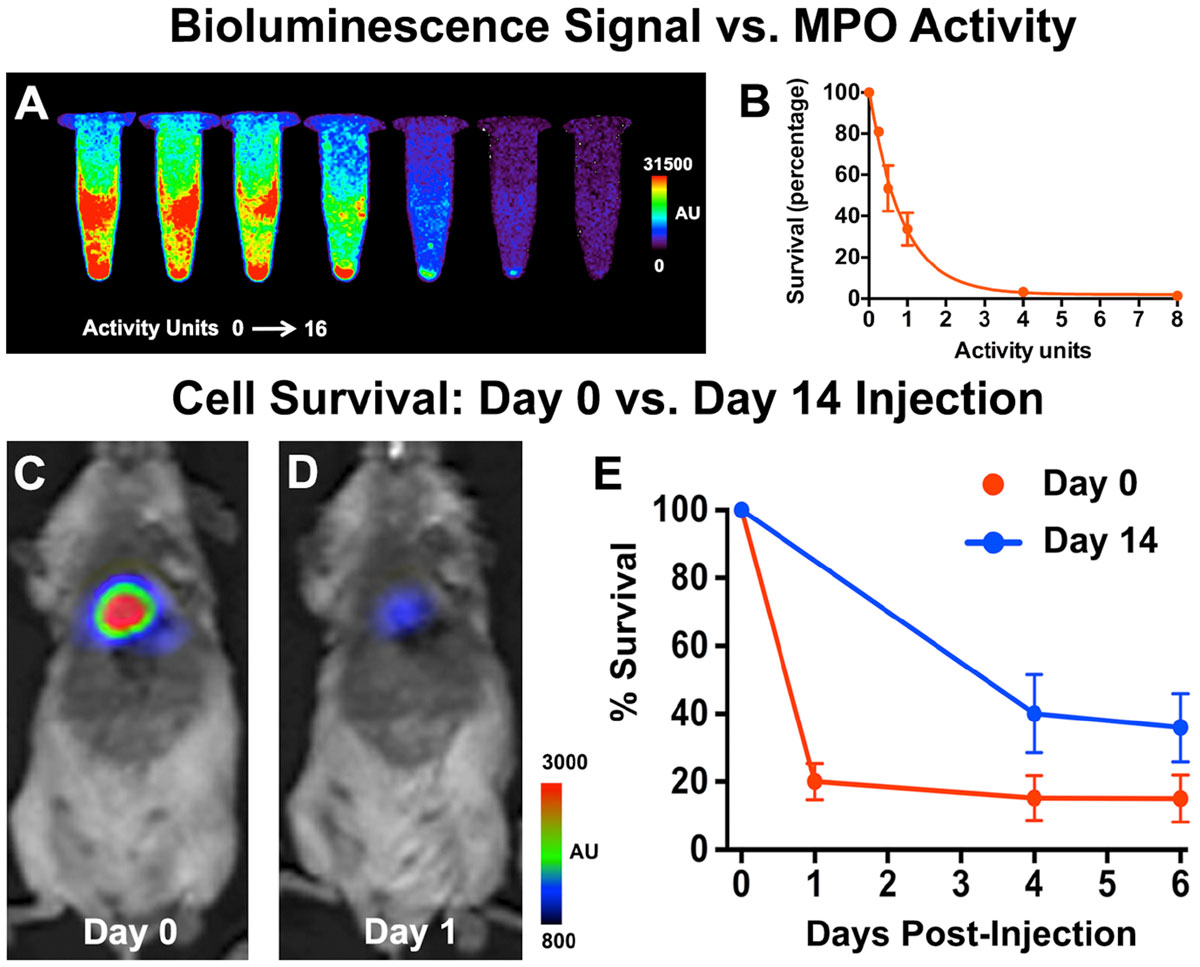
**Bioluminescence imaging of cell survival**. **A.** Cell viability decreases with increasing peroxidase activity in vitro. **B.** The presence of 3-8 peroxidase activity units for 30 minutes reduces cell survival to less than 20%. **C, D.** Cells injected into mice on Day 0 post ischemia show little viability by Day 1. **E.** In contrast, stem cells implanted on Day 14 post ischemic injury show significantly improved survival.

Within 1 hour of injection unbound Gd had washed out of the blood pool and myocardium. Activated Gd-MPO, however, was retained in the injured myocardium well beyond 2 hours (Figure 1). The concentration of activated probe ranged between 0.2 and 0.5 mM (Figure 1C), corresponding to 3-8 activity units of MPO. Cell phantoms (Figure 2A-B) revealed that this range of activity eliminated 80-100% of BLI signal (cell viability) within 30 minutes. Cells injected into mice on day 0 (Figure 2C-E), when MPO levels were high survived poorly. Survival was significantly improved (p < 0.05) when cells were injected into the myocardium on day 14, once MPO levels had dropped.

## Conclusions

MPO activity can be quantified *in vivo* using an activatable Gd probe. The levels of MPO encountered in healing ischemic myocardium are highly cytotoxic and are not compatible with the survival of injected cells. This has major implications for the use of cell therapy in acute ischemic injury.
